# Direct Position Determination of Multiple Non-Circular Sources with a Moving Coprime Array

**DOI:** 10.3390/s18051479

**Published:** 2018-05-08

**Authors:** Yankui Zhang, Bin Ba, Daming Wang, Wei Geng, Haiyun Xu

**Affiliations:** National Digital System Engineering and Technological Research R&D Center, Zhengzhou 450001, China; zhang_yk_2018@163.com (Y.Z.); wdm_wangdaming@163.com (D.W.); 18538116830@163.com (W.G.); xuhaiyun1995@sina.cn (H.X.)

**Keywords:** coprime array, direct position determination (DPD), non-circular sources, Cramer–Rao lower bound (CRLB)

## Abstract

Direct position determination (DPD) is currently a hot topic in wireless localization research as it is more accurate than traditional two-step positioning. However, current DPD algorithms are all based on uniform arrays, which have an insufficient degree of freedom and limited estimation accuracy. To improve the DPD accuracy, this paper introduces a coprime array to the position model of multiple non-circular sources with a moving array. To maximize the advantages of this coprime array, we reconstruct the covariance matrix by vectorization, apply a spatial smoothing technique, and converge the subspace data from each measuring position to establish the cost function. Finally, we obtain the position coordinates of the multiple non-circular sources. The complexity of the proposed method is computed and compared with that of other methods, and the Cramer–Rao lower bound of DPD for multiple sources with a moving coprime array, is derived. Theoretical analysis and simulation results show that the proposed algorithm is not only applicable to circular sources, but can also improve the positioning accuracy of non-circular sources. Compared with existing two-step positioning algorithms and DPD algorithms based on uniform linear arrays, the proposed technique offers a significant improvement in positioning accuracy with a slight increase in complexity.

## 1. Introduction

Passive localization technology applied to wireless signals is widely used in navigation, logistics management, smart homes, and the Internet of Things [1,2]. At present, there are two main passive localization technologies. The first is two-step location determination, which estimates location parameters, such as the angle and time delay, by constructing a mathematical model, and then obtains position coordinates using these parameters [3,4]. This is presented in an overview of two-step localization techniques [5], which provides a review of various fundamental methods, current trends, and state-of-the-art systems and algorithms employed in wireless position estimation. These two-step approaches are applicable to wireless local area networks, radio frequency identification, wireless sensor networks with ZigBee, Bluetooth technology, Bluetooth low energy, ultra-wide band, and the cellular system. The second passive localization technique is direct position determination (DPD), which directly establishes a mathematical model and computes the position of the target source using the observation station coordinates and received data, without the need for location parameter estimation [6,7]. In some commercial systems, directional antennas or sector antennas are used for positioning [8]. This paper mainly focuses on DPD research, using a coprime omnidirectional antenna array.

Direct position determination was first proposed by the scholar, A. J. Weiss in 2004 [9], who gave a basic model of DPD. At present, studies on DPD mainly consider single-station DPD based on angle, or multi-station DPD using the time delay or both the time delay and angle of the signals [9,10,11,12]. Single-station DPD based on angle is widely used because it requires no synchronization, and has a simple structure, low complexity, and low cost. DPD based on the angle of arrival with a moving station was proposed by Demissie in 2008 [13]. This mathematical model implements the Subspace Data Fusion (SDF) algorithm to improve the location performance. Oispuu et al. enhanced the estimation accuracy using the Capon iterative optimization and maximum likelihood (ML) method to improve the original position solution [14]. The above algorithms are based on circular sources (CS), however there are large numbers of non-circular sources (NS) in practical scenarios. Yin proposed a DPD technique based on a moving array [15] that fully leveraged the characteristics of NS to improve the positioning accuracy, and also derived the Cramer–Rao lower bound (CRLB) of this NS model. DPD algorithms for moving array tend to focus on uniform linear array, which results in low estimation accuracy because of the limited array freedom and array aperture. To date, there have been few reports on DPD techniques using coprime array.

Coprime arrays were developed from multiple input/multiple output radar, and are popular in array signal processing for radar, sonar, and radio astronomy [16,17]. Unlike uniform linear arrays, the spacing of coprime arrays can be greater than half the wavelength, and they have bigger array apertures, insignificant coupling effects, more degrees of freedom (DOF), and higher estimation accuracy [18,19]. Previous studies on position estimation with coprime arrays have mainly focused on two-step techniques, and the reported estimation accuracy is poor. Several papers have described the use of Bayesian learning to estimate the delay and angle with a single array based on coprime array models [20,21]. These methods demonstrate the idea of two-step location determination, but suffer from high complexity and low estimation precision.

In summary, existing positioning technologies based on uniform linear arrays suffer from low precision, whereas the two-step positioning technology based on coprime arrays has high complexity and lower accuracy than DPD algorithms. To improve the estimation accuracy for a moving array, this paper describes a coprime array technique for DPD, improves the SDF algorithm using the large aperture and high DOF of coprime arrays, and realizes high estimation precision of multiple sources. This algorithm is not only suitable for CS, but can also significantly improve the positioning precision of NS. This paper presents a detailed theoretical analysis and complexity comparison, and derives the CRLB of DPD based on the coprime array model. Simulation experiments show that, compared with the existing two-step localization algorithms and DPD algorithms with a moving uniform array, the proposed approach effectively improves the location precision in outdoor environments with a slight increase in complexity for both CS and NS.

The contributions of this paper are as follows:

(1) A coprime array is integrated into DPD. The physical model of DPD with a moving array is extended from a uniform array to a sparse non-uniform array, which effectively improves the positioning accuracy and enables the effective estimation of multiple NS.
(2) A virtual array model is constructed, and non-circular property is used to expand the array manifold, meaning the array DOF is greatly increased. This algorithm can not only realize the effective estimation in overdetermined conditions (the number of sensors in the array is bigger than the source number), but is also suitable for underdetermined conditions (the sensor number is smaller than the source number).
(3) The CRLB is derived under the proposed model, effectively proving that the proposed method achieves a significant decrease in the variance of position estimation.

The remainder of this paper is arranged as follows. Section 2 introduces the DPD model with a moving array. Section 3 describes the design of DPD for multiple NS with a moving coprime array. The CRLB for the model presented in this paper is derived in Section 4. Section 5 presents the results of performance simulation experiments, proving the validity of this algorithm. Section 6 gives the conclusions to this study. 

The following notation is used in this paper: $I_{N}$ and this represents the N-dimensional unit array. ${\left(•\right)}^{*}$, ${\left(•\right)}^{T}$ and ${\left(•\right)}^{H}$ represent the conjugate, transposition and conjugate transpose, respectively. The symbol ⊗ denotes the Kronecker product, and E(•) denotes the mathematical expectation.

## 2. DPD Model with a Moving Array

**Figure 1:**
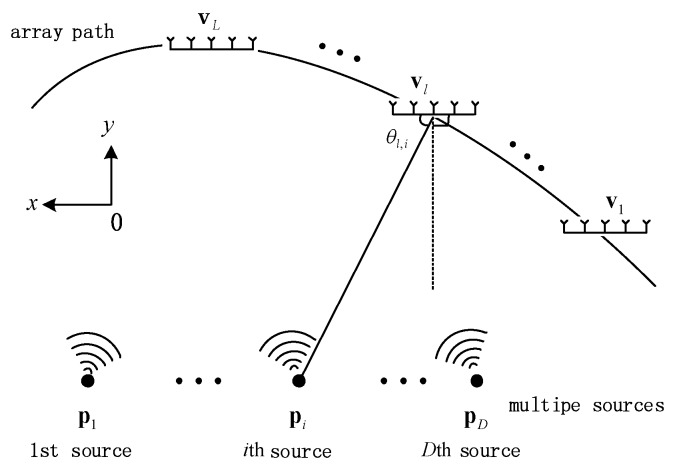
Geometry of one moving antenna array and multiple transmitters.

We assume that there are D stationary narrowband targets transmitting plane waves to the measuring station, and corresponding locations of the target signals are $p_{i}={\left(x_{i},y_{i}\right)}^{T}$, i∈{1,2,⋯,D}. Thus, the location vector of the (uncorrelated) source targets can be expressed as $p={\left(p_{1}^{T},p_{2}^{T}\cdots ,p_{D}^{T}\right)}^{T}$, and the sources are uncorrelated to each other. The receiving array at the observation station uses coprime array with M+N−1 sensors. All receiving sensors are omnidirectional antennas and have good phase consistency. According to the characteristics of the array, assume D<M+N−1, the observation station moved L positions during measurement, the observation position is $v_{l}={\left(x_{l},y_{l}\right)}^{T}$, and the number of snapshot at each observation position is K. The movement of the station is sufficiently slow that we can assume the channel environment does not change during measurements at the same observation position, and Doppler shift is not generated in this process. The received signal vector $r_{l}\left(k\right)$ represents the kth received snapshot at the lth observation position. Geometry of one moving antenna array and multiple transmitters is shown in Figure 1.

If we denote the kth received snapshot signal at the lth observation location as $s_{l}\left(k\right)$, and assume that the noise is additive Gaussian white noise, the received vector can be expressed as:

(1) $$r_{l}\left(k\right)=A_{l}\left(p\right)s_{l}\left(k\right)+n_{l}\left(k\right)$$

where the array manifold:

(2) $$A_{l}\left(p\right)=\left[\begin{array}{ccc} a_{l}\left(p_{1}\right) & \cdots & a_{l}\left(p_{D}\right) \end{array}\right]$$

and the steering vector:

(3) $$a_{l}\left(p_{i}\right)={\left[\begin{array}{ccc} e^{-j2\pi d_{1}\cos\theta_{l,i}/\lambda } & \cdots & e^{-j2\pi d_{M+N-1}\cos\theta_{l,i}/\lambda } \end{array}\right]}^{T}$$

$d_{j}$ is the distance from the jth antenna to the first antenna in the receiving array, $\cos\theta_{l,i}$ is the azimuthal cosine of the ith source for the lth observation position, which is similar to the parameter estimation part of two-step location. When the distance between the target and the array is far greater than the array aperture, the array is equivalent to a point and $\cos\theta_{l,i}$ can be replaced directly by the geometric relation between the position coordinates in DPD, that is:

(4) $$\cos\theta_{l,i}=\frac{\Delta x_{l,i}}{\left\|\Delta_{l,i}\right\|}=\frac{\left(x_{i}-x_{l}\right)}{\left\|\left(x_{i}-x_{l},y_{i}-y_{l}\right)\right\|}$$

## 3. Proposed Algorithm

### 3.1. DPD Model Based on Coprime Array

**Figure 2:**
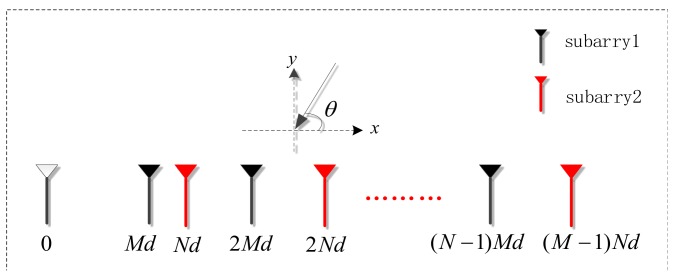
Geometry of coprime array.

A coprime array is a special type of sparse array composed of two sparse uniform arrays in which the sensor spacings are coprime integer multiples of half the wavelength. Figure 2 illustrates the coprime array model, where d=λ/2, and λ is the signal wavelength. Subarray 1 contains N array sensors with sensor spacing Md, and subarray 2 contains M array sensors with sensor spacing Nd. The two subarrays are on the same line, and the first sensors in each array coincide, so the whole array contains M+N−1 array sensors. $d_{j}$ is the location of the jth array sensor, and

(5) $$d_{j}\in \left\{0,Md\cdots M(N-1)d\right\}\cup \left\{Nd\cdots N(M-1)d\right\}$$

According to the sensor distance in each subarray, we can obtain the self-difference $S_{self-diff1}$, $S_{self-diff2}$ and the cross-difference $S_{cross-diff}$ of the two sparse arrays. These differences represent the relative position of any two elements, and form the basis of a virtual array. The detailed concept of a virtual array is discussed in References [3,4,17,19]. The self-differences and cross-difference are calculated as

(6) $$S_{self-diff1}=\{Mn_{1}-Mn_{2},0\leq n_{1},n_{2}\leq N-1\}$$

(7) $$S_{self-diff2}=\{Nm_{1}-Nm_{2},1\leq m_{1},m_{2}\leq M-1\}$$

(8) $$S_{cross-diff}=\{\pm \left(Nm-Mn\right),1\leq m\leq M-1,0\leq n\leq N-1\}$$

**Figure 3:**
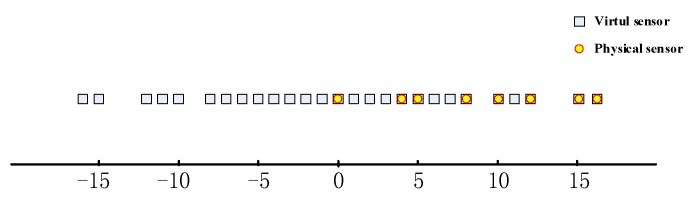
Position distribution of physical sensors and virtual sensors (M=4, N=5).

According to (6)–(8), the virtual array structure will be obtained by removing repeated virtual array sensors. For M=4 and N=5, the real sensors of subarrays are those with numbers {0,4,8,12,16} and {0,5,10,15}, respectively, and the virtual sensor locations are {0,±1,±2,±3,±4,±5,±6,±7,±8,±10,±11,±12,±15,±16}. The distribution of physical array sensors and virtual array sensors are shown in Figure 3. Obviously, coprime array structure has an increased array aperture and greater array DOF. 

### 3.2. MCA-DPD of Multiple Non-Circular Sources Using a Moving Coprime Array

Traditionally, sources can be divided into circular sources and non-circular sources by the second-order statistical properties. The most important difference between circular and non-circular sources is the ellipse covariance. Circular sources refer to those whose elliptical covariance is zero, and non-circular sources refer to those in which elliptical covariance is nonzero. Traditional NS include binary phase shift keying (BPSK), aryamplitude shift keying (ASK) and pulse amplitude modulation (PAM) sources. As the elliptical covariance of non-circular sources is nonzero, the proposed method makes full use of this characteristic, to improve the estimation accuracy based on a coprime array.

Firstly, the kth snapshot of the NS for the lth observation location can be expressed as

(9) $$s_{l}\left(k\right)={\left[\begin{array}{cccc} s_{l,1}^{}\left(k\right) & s_{l,2}^{}\left(k\right) & \cdots & s_{l,D}^{}\left(k\right) \end{array}\right]}^{T}$$

where $s_{l,i}^{}\left(k\right)$ represents the kth snapshot data for the ith source at the lth observation location. As the transmitted signal phase for the same signal source is fixed in non-circular signals, we have:

(10) $$s_{l,i}^{}\left(k\right)=s_{l,0}^{\left(i\right)}\left(k\right)e^{j\varphi_{i}}$$

where $\varphi_{i}$ indicates the ith phase of the sent signal and, $s_{l,0}^{\left(i\right)}\left(k\right)$ is a real number representing the signal amplitude. Thus, we can write:

(11) $$s_{l}\left(k\right)=\left[\begin{array}{c} s_{l,1}^{}\left(k\right)\\ s_{l,2}^{}\left(k\right)\\ \vdots \\ s_{l,D}^{}\left(k\right) \end{array}\right]=\left[\begin{array}{c} s_{l,0}^{\left(1\right)}\left(k\right)e^{j\varphi_{1}}\\ s_{l,0}^{\left(2\right)}\left(k\right)e^{j\varphi_{2}}\\ \vdots \\ s_{l,0}^{\left(D\right)}\left(k\right)e^{j\varphi_{D}} \end{array}\right]=\left[\begin{array}{cccc} e^{j\varphi_{1}} & 0 & \cdots & 0\\ 0 & e^{j\varphi_{2}} & \ddots & \vdots \\ \vdots & \ddots & \ddots & 0\\ 0 & \cdots & 0 & e^{j\varphi_{D}} \end{array}\right]\left[\begin{array}{c} s_{l,0}^{\left(1\right)}\left(k\right)\\ s_{l,0}^{\left(2\right)}\left(k\right)\\ \vdots \\ s_{l,0}^{\left(D\right)}\left(k\right) \end{array}\right]=\Phi s_{l,0}\left(k\right)$$

(12) $$\Phi =\left[\begin{array}{cccc} e^{j\varphi_{1}} & 0 & \cdots & 0\\ 0 & e^{j\varphi_{2}} & \ddots & \vdots \\ \vdots & \ddots & \ddots & 0\\ 0 & \cdots & 0 & e^{j\varphi_{D}} \end{array}\right]$$

(13) $$s_{l,0}\left(k\right)={\left[\begin{array}{cccc} s_{l,0}^{\left(1\right)}\left(k\right) & s_{l,0}^{\left(2\right)}\left(k\right) & \cdots & s_{l,0}^{\left(D\right)}\left(k\right) \end{array}\right]}^{T}$$

where $s_{l,0}\left(k\right)$ is a real vector. According to these expressions, the kth received snapshot at the lth observation position is expressed as:

(14) $$r_{l}\left(k\right)=A_{l}\left(p\right)s_{l}\left(k\right)+n_{l}\left(k\right)=A_{l}\left(p\right)\Phi s_{l,0}\left(k\right)+n_{l}\left(k\right)$$

Define the receiving signals as:

(15) $$z_{l}\left(k\right)=\left[\begin{array}{c} r_{l}\left(k\right)\\ r_{l}^{*}\left(k\right) \end{array}\right]=\left[\begin{array}{c} A_{l}\left(p\right)s_{l}\left(k\right)\\ A_{l}^{*}\left(p\right)s_{l}^{*}\left(k\right) \end{array}\right]+\left[\begin{array}{c} n_{l}\left(k\right)\\ n_{l}^{*}\left(k\right) \end{array}\right]$$

using:

(16) $$s_{l}^{*}\left(k\right)=\Phi_{}^{*}s_{l,0}^{*}\left(k\right)=\Phi_{}^{*}\Phi_{}^{-1}s_{l}^{}\left(k\right)={\left(\Phi_{}^{*}\right)}^{2}s_{l}^{}\left(k\right)$$

we find that:

(17) $$z_{l}\left(k\right)=\left[\begin{array}{c} r_{l}\left(k\right)\\ r_{l}^{*}\left(k\right) \end{array}\right]=\left[\begin{array}{c} A_{l}\left(p\right)\\ A_{l}^{*}\left(p\right)\Phi_{}^{*}\Phi_{}^{*} \end{array}\right]s_{l}\left(k\right)+\left[\begin{array}{c} n_{l}\left(k\right)\\ n_{l}^{*}\left(k\right) \end{array}\right]=B_{l}\left(p\right)s_{l}\left(k\right)+\left[\begin{array}{c} n_{l}\left(k\right)\\ n_{l}^{*}\left(k\right) \end{array}\right]$$

where:

(18) $$B_{l}\left(p\right)=\left[\begin{array}{c} A_{l}\left(p\right)\\ A_{l}^{*}\left(p\right)\Phi_{}^{*}\Phi_{}^{*} \end{array}\right]=\left[\begin{array}{cccc} b_{l}\left(p_{1}\right) & b_{l}\left(p_{2}\right) & \cdots & b_{l}\left(p_{D}\right) \end{array}\right]$$

(19) $$b_{l}\left(p_{i}\right)=\left[\begin{array}{c} a_{l}\left(p_{i}\right)\\ a_{l}^{*}\left(p_{i}\right)e^{-j2\varphi_{i}} \end{array}\right]$$

The received covariance matrix for each measurement position is as follows:

(20) $$\begin{array}{cc} R_{l} & =\frac{1}{K}\sum_{k=1}^{K}z_{l}\left(k\right)z_{l}^{H}\left(k\right)\\ & =\sum_{i=1}^{D}\sigma_{l,i}^{2}b_{l}\left(p_{i}\right)b_{l}^{H}\left(p_{i}\right)+\sigma_{n}^{2}I \end{array}$$

where $\sigma_{l,i}^{2}$ denotes the power of the ith transmitted signal at the lth observation position and $\sigma_{n}^{2}$ denotes noise power. In order to make full use of the sparse characteristic of the coprime array, we vectorize the received covariance matrix

(21) $$\begin{array}{cc} z_{l} & =vec\left(R_{l}\right)\\ & =vec\left(\sum_{i=1}^{D}\sigma_{l,i}^{2}b_{l}\left(p_{i}\right)b_{l}^{H}\left(p_{i}\right)\right)+\sigma_{n}^{2}{\bar{I}}_{}\\ & =H_{l}\left(p\right)\mu +\sigma_{n}^{2}{\bar{I}}_{} \end{array}$$

where μ is the signal power vector, with

(22) $$H_{l}\left(p\right)=\left[b_{l}^{*}\left(p_{1}\right)⊗b_{l}^{}\left(p_{1}\right),b_{l}^{*}\left(p_{2}\right)⊗b_{l}^{}\left(p_{2}\right),\cdots b_{l}^{*}\left(p_{D}\right)⊗b_{l}^{}\left(p_{D}\right)\right]$$

(23) $$\bar{I}=vec\left(I_{M+N-1}\right)$$

The elements of $b_{l}^{*}\left(p_{i}\right)⊗b_{l}^{}\left(p_{i}\right)$ can be expressed as $e^{\pm j2\pi d_{j}\cos\theta_{l,i}/\lambda }$, $e^{\pm j2\pi (d_{j}-d_{c})\cos\theta_{l,i}/\lambda }$, where $d_{j},d_{c}\in \left\{0,Md\cdots M(N-1)d\right\}\cup \left\{Nd\cdots N(M-1)d\right\}$. According to the concepts of difference and virtual arrays, $H_{l}\left(p\right)$ contains repeated row vectors, ${\overset{⌣}{H}}_{l}\left(p\right)$ is the continuous response part in the virtual array manifold, the corresponding response of the virtual array is ${\overset{⌣}{z}}_{l}$, and noise column vector is $\sigma_{n}^{2}{\overset{⌣}{e}}_{l}$. Hence,

(24) $${\overset{⌣}{z}}_{l}={\overset{⌣}{H}}_{l}\left(p\right)\mu +\sigma_{n}^{2}{\overset{⌣}{e}}_{l}$$

**Figure 4:**
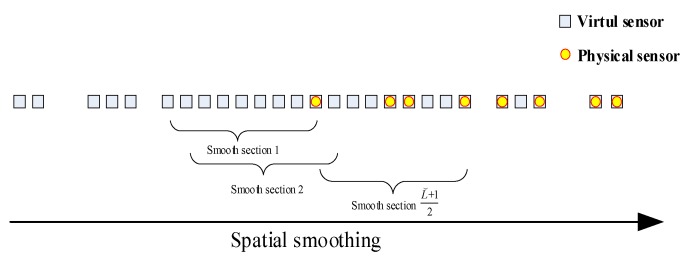
Spatial smoothing of the virtual array.

We apply spatial smoothing to ${\overset{⌣}{z}}_{l}$, as shown in Figure 4, and ${\overset{⌣}{z}}_{l}$ has a conjugate symmetric distribution, so we set the length of the continuous virtual array to $\overset{⌣}{L}$, and take the length of the smooth segment to be $\left(\overset{⌣}{L}+1\right)/2$. The position of the sth smooth subarray is ${\overset{⌣}{z}}_{l,s}$, and the noise vector is $\sigma_{n}^{2}{\overset{⌣}{e}}_{l,s}$. For the virtual array

(25) $$\left\{\left(-s+n-1\right)d,\;n=0,1,\cdots \left(\overset{⌣}{L}-1\right)/2\right\}$$

(26) $$\begin{array}{cc} {\overset{⌣}{z}}_{l,s} & ={\overset{⌣}{H}}_{l,s}\left(p\right)\mu +\sigma_{n}^{2}{\overset{⌣}{e}}_{l,s}\\ & ={\overset{⌣}{H}}_{l,1}\left(p\right)\Psi^{s-1}\mu +\sigma_{n}^{2}{\overset{⌣}{e}}_{l,s} \end{array}$$

with:

(27) $$\Psi =\left(\begin{array}{cccc} e^{-j\pi \frac{\left(x_{1}-x_{l}\right)}{\left\|\left(x_{1}-x_{l},y_{1}-y_{l}\right)\right\|}} & & & \\ & e^{-j\pi \frac{\left(x_{2}-x_{l}\right)}{\left\|\left(x_{2}-x_{l},y_{2}-y_{l}\right)\right\|}} & & \\ & & \ddots & \\ & & & e^{-j\pi \frac{\left(x_{D}-x_{l}\right)}{\left\|\left(x_{D}-x_{l},y_{D}-y_{l}\right)\right\|}} \end{array}\right)$$

(28) $${\overset{⌣}{H}}_{l,1}\left(p\right)=\left(\begin{array}{cccc} 1 & 1 & \cdots & 1\\ h_{l}\left(p_{1}\right) & h_{l}\left(p_{2}\right) & \cdots & h_{l}\left(p_{D}\right)\\ \vdots & \vdots & \vdots & \vdots \\ {\left[h_{l}\left(p_{1}\right)\right]}^{\frac{\overset{⌣}{L}-1}{2}} & {\left[h_{l}\left(p_{2}\right)\right]}^{\frac{\overset{⌣}{L}-1}{2}} & \cdots & {\left[h_{l}\left(p_{D}\right)\right]}^{\frac{\overset{⌣}{L}-1}{2}} \end{array}\right)$$

$h_{l}\left(p_{i}\right)=e^{-j\pi \frac{\left(x_{i}-x_{l}\right)}{\left\|\left(x_{i}-x_{l},y_{i}-y_{l}\right)\right\|}}$, and $h_{l}\left(p_{i}\right)={\left[\begin{array}{cccc} 1 & h_{l}\left(p_{i}\right) & \cdots & {\left[h_{l}\left(p_{i}\right)\right]}^{\frac{\overset{⌣}{L}-1}{2}} \end{array}\right]}^{T}$. Eigenvalue decomposition (EVD) is carried out based on the weighted mean for all the autocorrelation matrices of the smooth subarray, thus:

(29) $$\begin{array}{cc} {\overset{⌣}{R}}_{l} & ={\left(\frac{\overset{⌣}{L}+1}{2}\right)}^{-1}\sum_{s=1}^{\frac{\overset{⌣}{L}+1}{2}}{\overset{⌣}{z}}_{l,s}{\overset{⌣}{z}}_{l,s}^{H}\\ & =\left[\begin{array}{cc} U_{l}^{\left(s\right)} & U_{l}^{\left(n\right)} \end{array}\right]\Sigma {\left[\begin{array}{cc} U_{l}^{\left(s\right)} & U_{l}^{\left(n\right)} \end{array}\right]}^{H} \end{array}$$

Take $U_{l}^{\left(n\right)}=\left[\begin{array}{c} U_{l,1}^{\left(n\right)}\\ U_{l,2}^{\left(n\right)} \end{array}\right]$, where $U_{l,1}^{\left(n\right)}$ and $U_{l,2}^{\left(n\right)}$ are matrices with the same dimensions. Through the above deduction;

(30) $$U_{l,1}^{\left(n\right)}={\left(U_{l,2}^{\left(n\right)}\right)}^{*}$$

Furthermore, ${\left(U_{l,1}^{\left(n\right)}\right)}^{T}={\left(U_{l,2}^{\left(n\right)}\right)}^{H}$, where $U_{l,2}^{\left(n\right)}{\left(U_{l,1}^{\left(n\right)}\right)}^{H}$ and $U_{l,1}^{\left(n\right)}{\left(U_{l,2}^{\left(n\right)}\right)}^{H}$ are conjugated relations. Considering the location estimation expressions for NS in [15], we obtain the SDF objective function for NS as

(31) $$f\left(p\right)=\sum_{l=1}^{L}\{{\left(h_{l}\left(p\right)\right)}^{H}U_{l,1}^{\left(n\right)}{\left(U_{l,1}^{\left(n\right)}\right)}^{H}h_{l}\left(p\right)\;-\left|{\left(h_{l}\left(p\right)\right)}^{T}U_{l,2}^{\left(n\right)}{\left(U_{l,1}^{\left(n\right)}\right)}^{H}h_{l}\left(p\right)\right|\}$$

The target source locations can then be obtained through a spectral peak search of D minimum points.

### 3.3. Algorithm Steps Conclusion

Many steps of this algorithm can be summarized as follow:

Step 1: Construct a DPD model with a single moving array according to the coprime array structure.
Step 2: Vectorize the covariance matrix of each observation position, and then construct the virtual array model based on the continuous virtual response using Equations (21)–(24).
Step 3: Apply spatial smoothing to the continuous virtual array response, and perform EVD on the weighted smoothing subarray according to Equation (29).
Step 4: Establish the cost function of the target position using SDF. Using Equation (31), the D minimum points can be obtained via a spectral peak search, giving the locations of the target sources.

**Figure 5:**
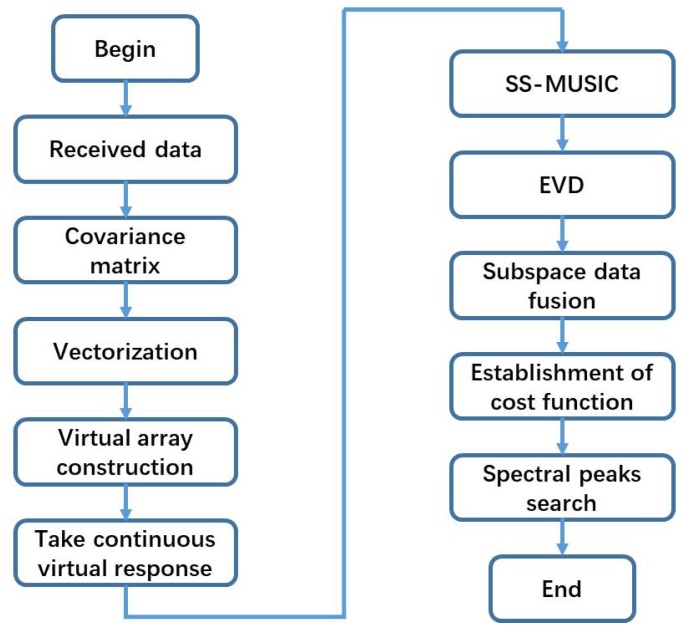
Flowchart of the proposed algorithm.

A detailed flowchart of this algorithm is shown in Figure 5.

Furthermore, the DPD algorithm, based on a moving coprime array (MCA-DPD) of this paper for CS and NS, are similar. Indeed, the main difference is the use of non-circular characteristics and the establishment of the cost function, as Equations (17) and (31).

## 4. Performance Analysis

### 4.1. Derivation of the CRLB

As the lower bound of the unbiased estimation variance, the CRLB represents the degree of parameter estimation deviation. For MCA-DPD with multiple NS, the CRLB is given according to References [22,23,24]. Firstly, the kth received signal snapshot vector for all observation locations is:

(32) $$z\left(k\right)={\left[z_{1}^{T}\left(k\right),\cdots z_{L}^{T}\left(k\right)\right]}^{T}$$

The corresponding transmission signal vector and the noise vector are expressed as:

(33) $$s\left(k\right)={\left[s_{1}^{T}\left(k\right),\cdots s_{L}^{T}\left(k\right)\right]}^{T}$$

(34) $$n\left(k\right)={\left[n_{1}^{T}\left(k\right),\cdots n_{L}^{T}\left(k\right)\right]}^{T}$$

The array manifold B is:

(35) $$B=\left[\begin{array}{ccc} B_{l}\left(p\right) & \cdots & 0\\ \vdots & \ddots & \vdots \\ 0 & \cdots & B_{L}\left(p\right) \end{array}\right]$$

Hence, the receiving signal can be expressed as:

(36) z(k)=Bs(k)+n(k)

where the noise vector n(k) follows the complex Gaussian distribution:

(37) n(k)=z(k)−Bs(k)

(38) $$P\left(z\left(1\right),\cdots ,z\left(K\right)\right)=\frac{1}{{\left(2\pi \right)}^{\left(M+N-1\right)K}{\left(\sigma_{n}^{2}/2\right)}^{\left(M+N-1\right)K}}\exp^{-\frac{1}{\sigma_{n}^{2}}\sum_{k=1}^{K}{\left[z\left(k\right)-Bs\left(k\right)\right]}^{H}\left[z\left(k\right)-Bs\left(k\right)\right]}$$

Taking the logarithm, the log-likelihood function can be obtained as:

(39) $$\begin{array}{cc} L\left(z\left(1\right),\cdots ,z\left(K\right)\right)= & -\left(M+N-1\right)K\ln\left(2\pi \right)-\left(M+N-1\right)K\ln\left(\sigma_{n}^{2}/2\right)\\ & -\frac{1}{\sigma_{n}^{2}}\sum_{k=1}^{K}{\left[z\left(k\right)-Bs\left(k\right)\right]}^{H}\left[z\left(k\right)-Bs\left(k\right)\right] \end{array}$$

Let $\bar{s}\left(k\right)$ and $\tilde{s}\left(k\right)$ be the real and imaginary parts of s(k), respectively, i.e., s¯(k)=Re[s(k)], s˜(k)=Im[s(k)]. The impulse function can be expressed as:

(40) $$\delta \left(t\right)=\{\begin{array}{cc} 1 & t=0\\ 0 & t\neq 0 \end{array}$$

The second-order moment of origin and the mixed second-order moment of the log-likelihood function for $\sigma_{n}^{2}$, s(k) and p are as follows:

(41) $$\frac{\partial L}{\partial \sigma_{n}^{2}}=-\frac{\left(M+N-1\right)K}{\sigma_{n}^{2}}+\frac{1}{\sigma_{n}^{4}}\sum_{k=1}^{K}n_{}^{H}\left(k\right)n\left(k\right)$$

(42) $$E\left[{\left(\frac{\partial L}{\partial \sigma_{n}^{2}}\right)}^{2}\right]=\frac{\left(M+N-1\right)K}{\sigma_{n}^{4}}$$

(43) ∂L∂s¯(k)=2σn2Re[BHn(k)]

(44) ∂L∂s˜(k)=2σn2Im[BHn(k)]

(45) E[(∂L∂s¯(k))(∂L∂s¯(q))T]=2σn2Re[BHB]δ(k−q)

(46) E[(∂L∂s˜(k))(∂L∂s˜(q))T]=2σn2Im[BHB]δ(k−q)

(47) E[(∂L∂s¯(k))(∂L∂s˜(q))T]=−2σn2Im[BHB]δ(k−q)

Computing $\frac{\partial L}{\partial x_{i}}$ and $\frac{\partial L}{\partial y_{i}}$ as:

(48) ∂L∂xi=2σn2∑k=1KRe(sH(k)∂BH∂xin(k))

(49) ∂L∂yi=2σn2∑k=1KRe(sH(k)∂BH∂yin(k))

we can write $\frac{\partial L}{\partial p}$ as:

(50) ∂L∂p=2σn2∑k=1KRe(FH(k)DHn(k))

where $F\left(k\right)=I_{2}⊗diag\left(s\left(k\right)\right)$, $I_{2}=\left[\begin{array}{cc} 1 & 0\\ 0 & 1 \end{array}\right]$, and $D=\left[\begin{array}{ccccc} \frac{\partial B^{H}}{\partial x_{1}} & \frac{\partial B^{H}}{\partial y_{1}} & \cdots & \frac{\partial B^{H}}{\partial x_{D}} & \frac{\partial B^{H}}{\partial y_{D}} \end{array}\right]$. Thus,

(51) $$E\left[\left(\frac{\partial L}{\partial \sigma_{n}^{2}}\right){\left(\frac{\partial L}{\partial p}\right)}^{T}\right]=0$$

(52) E[(∂L∂s¯(k))(∂L∂p)T]=2σn2∑k=1KRe[BHDF(k)]

(53) E[(∂L∂s˜(k))(∂L∂p)T]=2σn2∑k=1KIm[BHDF(k)]

(54) E[(∂L∂p)(∂L∂p)T]=2σn2∑k=1KRe[FH(k)DDHF(k)]

The Fisher information matrix $\Omega ={\left[E\left(\chi \chi^{T}\right)\right]}^{-1}$, where:

(55) $$\chi^{T}=\partial L/\partial \left[\begin{array}{ccccccc} \sigma_{n}^{2} & {\bar{s}}_{}^{T}\left(1\right) & {\tilde{s}}_{}^{T}\left(1\right) & \cdots & {\bar{s}}_{}^{T}\left(K\right) & {\tilde{s}}_{}^{T}\left(K\right) & p^{T} \end{array}\right]$$

Finally, the CRLB expression of the multiple NS can be obtained from the Fisher information matrix as

(56) CRLB(p)=σn22{∑k=1KRe[FH(k)DHPB⊥DF(k)]}−1

where $P_{B}^{⊥}=I-P_{B}^{}=I-B{\left(B^{H}B\right)}^{-1}B^{H}$.

This gives the CRLB for NS. The CRLB for CS is similar, with the main difference being the structure of the array manifold. The CRLB for CS is:

(57) CRLB(p)=σn22{∑k=1KRe[FH(k)DHPA⊥DF(k)]}−1

with $P_{A}^{⊥}=I-P_{A}^{}=I-A{\left(A^{H}A\right)}^{-1}A^{H}$, and $A=\left[\begin{array}{ccc} A_{l}\left(p\right) & \cdots & 0\\ \vdots & \ddots & \vdots \\ 0 & \cdots & A_{L}\left(p\right) \end{array}\right]$.

### 4.2. Complexity Analysis

In determining the computational complexity of this algorithm, we focus on the individual parts of the algorithm. Solving the received covariance matrix for each observation position has a computational complexity of $O\left(LK{\left(M+N-1\right)}^{2}\right)$ for CS and $O\left(4LK{\left(M+N-1\right)}^{2}\right)$ for NS. For the response of the virtual array, the computational complexity of spatial smoothing and the weighted covariance matrix is $O\left(L{\left(\frac{\overset{⌣}{L}+1}{2}\right)}^{3}\right)$ for CS; there are twice as many continuous virtual elements in the NS case, so the computational complexity is $O\left(8L{\left(\frac{\overset{⌣}{L}+1}{2}\right)}^{3}\right)$ for NS.

**Figure 6:**
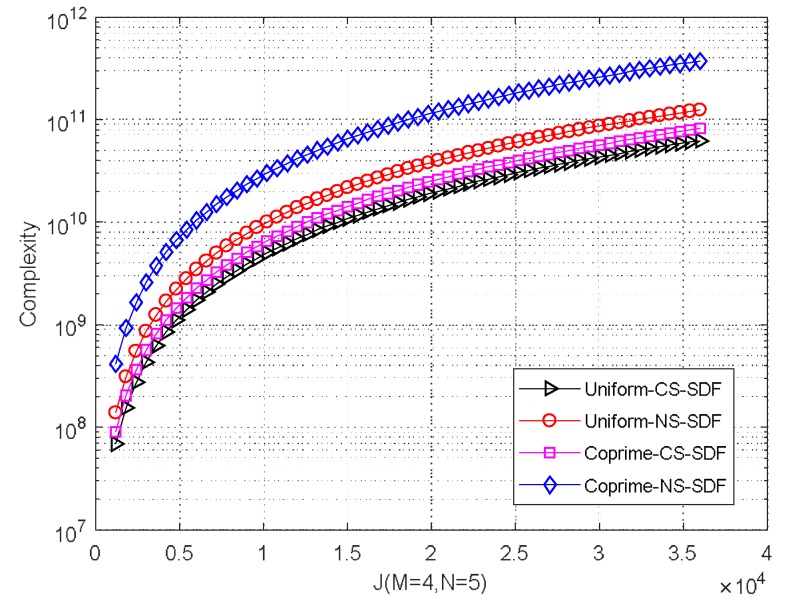
Complexity comparison with different grid number. Uniform-CS-SDF: SDF for CS based on uniform array; Uniform-NS-SDF: SDF for NS based on uniform array; Coprime-CS-SDF (proposed): SDF for CS based on coprime array; Coprime-NS-SDF (proposed): SDF for NS based on coprime array.

The weighted objective function is constructed by applying EVD to the weighted covariance matrix and SDF. The computational complexity of these operations is $O\left(J^{2}\frac{\overset{⌣}{L}+1}{2}\left(\frac{\overset{⌣}{L}+1}{2}-D\right)\right)$ for CS and $O\left(2J^{2}\frac{\overset{⌣}{L}+1}{2}\left(2\frac{\overset{⌣}{L}+1}{2}-D\right)\right)$ for NS, where J represents the number of grid points in a two-dimensional search. Overall, the computational complexity of the proposed MCA-DPD algorithm is $O\left(L{\left(\frac{\overset{⌣}{L}+1}{2}\right)}^{3}+\;J^{2}{\left(\frac{\overset{⌣}{L}+1}{2}\right)}^{2}-DJ^{2}\frac{\overset{⌣}{L}+1}{2}+LK{\left(M+N-1\right)}^{2}\right)$ for CS and $O\left(8L{\left(\frac{\overset{⌣}{L}+1}{2}\right)}^{3}+4J^{2}{\left(\frac{\overset{⌣}{L}+1}{2}\right)}^{2}-2DJ^{2}\frac{\overset{⌣}{L}+1}{2}+4LK{\left(M+N-1\right)}^{2}\right)$ for NS. Using a uniform array, the computational complexity of SDF is $O\left(L{\left(M+N-1\right)}^{3}+\left(LK+J^{2}\right){\left(M+N-1\right)}^{2}-J^{2}D\left(M+N-1\right)\right)$ for CS and $O\left(8L{\left(M+N-1\right)}^{3}+\left(4LK+2J^{2}\right){\left(M+N-1\right)}^{2}-2J^{2}D\left(M+N-1\right)\right)$ for NS. A comparison of the complexity for different grid sizes is shown in Figure 6. When M=4, N=5, K=100, the number of observed positions is L=3, and the number of spatial smoothing segment is $\frac{\overset{⌣}{L}+1}{2}=9$. Clearly, the complexity of the proposed algorithm is slightly higher because of the coprime array model, and the complexity of DPD is strongly influenced by the size of the search grid.

## 5. Simulation Results

In this section, we present the results of simulation experiments to examine the performance of MCA-DPD with multiple sources. These results are compared with those from a two-step localization algorithm and the basic SDF algorithm. To measure the positioning accuracy of this algorithm, we define the root mean square error (RMSE) as

(58) $$RMSE=\sqrt{\frac{1}{QD}\sum_{m=1}^{Q}\sum_{i=1}^{D}{\left\|{\hat{p}}_{i}\left(m\right)-p_{i}\right\|}^{2}}$$

where Q is the number of Monte Carlo simulations, D is the number of source targets, and ${\hat{p}}_{i}\left(m\right)$ is the ith source location in the mth Monte Carlo simulation. The simulation conditions are listed in Table 1.

**Simulation** **1.** *Positioning performance of the proposed algorithm at different signal-to-noise ratios (SNRs).*

**Figure 7:**
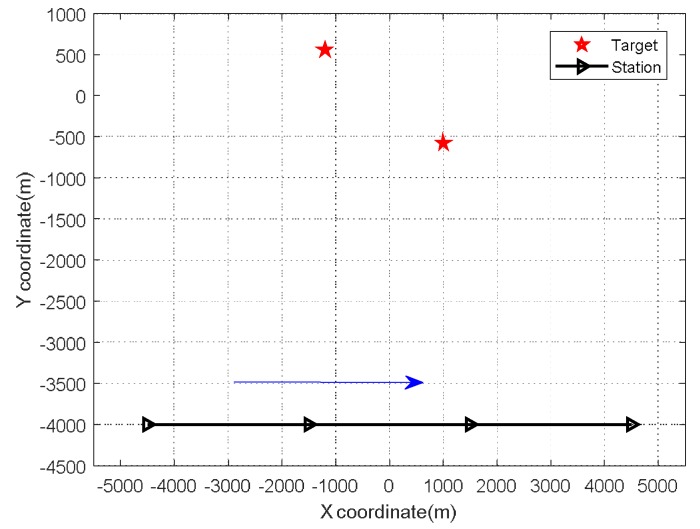
Trajectory of the station movement related to the target sources.

**Figure 8:**
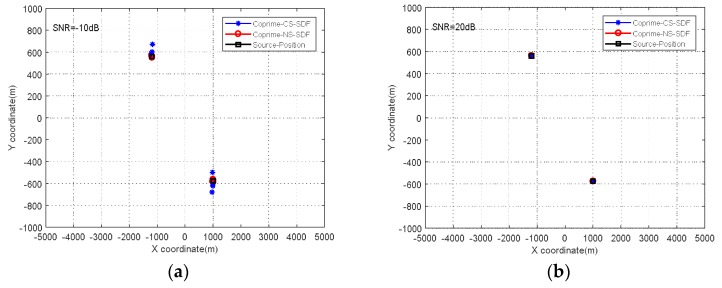
Positioning results of the proposed algorithm (**a**) *SNR* = −10 dB (**b**) *SNR* = 20 dB.

In this experiment, the observation positions were (−4500, −4000), (−1500, −4000), (1500, −4000), and (4500, −4000), and the trajectory of the station movement in relation to the target sources, was as shown in Figure 7. To verify the performance of the proposed algorithm under different noise levels, we applied the MCA-DPD algorithm with *SNRs* of −10 dB and 20 dB. The simulation results in Figure 8 show that, for both CS and NS, MCA-DPD achieves better estimation performance with higher *SNR,* and can effectively estimate the locations of source targets at lower *SNR*.

**Simulation** **2.** *RMSE comparison of MCA-DPD, SDF, two-step localization, and CRLB under different SNRs.*

**Figure 9:**
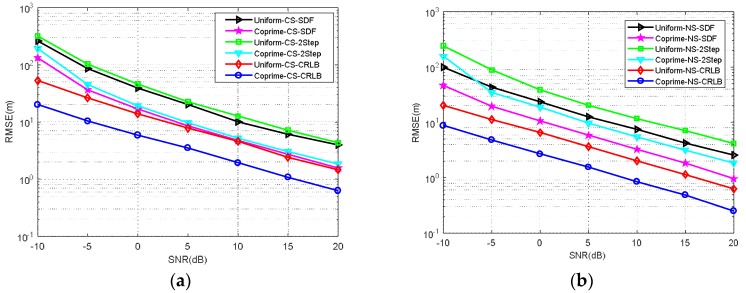
RMSE performance comparison under different *SNR* (**a**) circular sources (**b**) non-circular sources. Uniform-CS-2Step/Uniform-NS-2step: two-step localization for CS/NS based on uniform array; Coprime-CS-2Step/Coprime-NS-2step: two-step localization for CS/NS based on coprime array; Uniform-CS-CRLB/Uniform-NS-CRLB: CRLB for CS/NS based on uniform array; Coprime-CS-CRLB/Coprime-NS-CRLB: CRLB for CS/NS based on coprime array.

This simulation measured the RMSE of SDF, two-step localization, and MCA-DPD. The estimation performance of two-step localization based on the coprime array, and the CRLB based on a uniform linear array and the coprime array, was also examined. Figure 9a shows the results from the SDF algorithm, with a uniform array and MCA-DPD, the two-step positioning performance, and the CRLB comparison for the CS case. Figure 9b compares the performance of these methods for the NS case. When *SNR* = 0 dB, the positioning accuracy improves by ~20 m in the NS case, and when *SNR* = 20 dB, the positioning accuracy improves by ~2 m. The two-step localization algorithm is less accurate than DPD under the same simulation conditions, and the coprime array model is more accurate than the uniform array model in the same location algorithm. Furthermore, the CRLB of the coprime array model is obviously lower than that of the uniform linear array. The positioning accuracy is effectively improved by the use of the coprime array in both two-step localization and SDF.

**Simulation** **3.** *RMSE comparison of MCA-DPD, SDF, two-step localization, and the CRLB under different snapshot numbers.*

**Figure 10:**
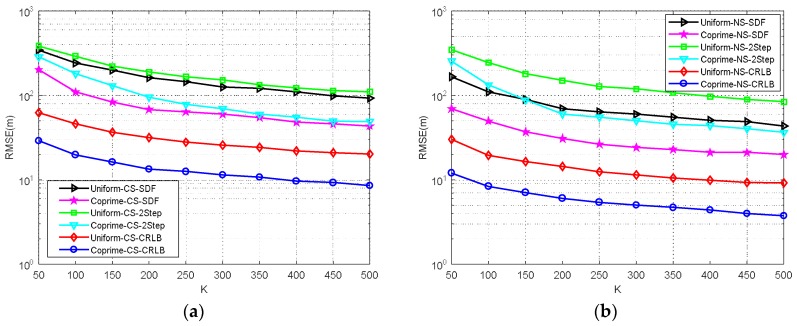
Estimation performance of different snapshots (**a**) CS (**b**) NS.

The number of snapshots is an important factor in the positioning accuracy. This simulation compared the performance of MCA-DPD, two-step localization, SDF, and CRLB for CS and NS under a uniform array and coprime array, with different numbers of snapshots and *SNR* = −10 dB. Figure 10 shows that, as the number of snapshots increases, the RMSE performance effectively improves in both the CS and NS cases. DPD outperforms two-step localization for the same array model, and the coprime array is better than the uniform array for each positioning algorithm.

**Simulation** **4.** *RMSE comparison of MCA-DPD, SDF, two-step localization, and the CRLB under different trajectories.*

**Figure 11:**
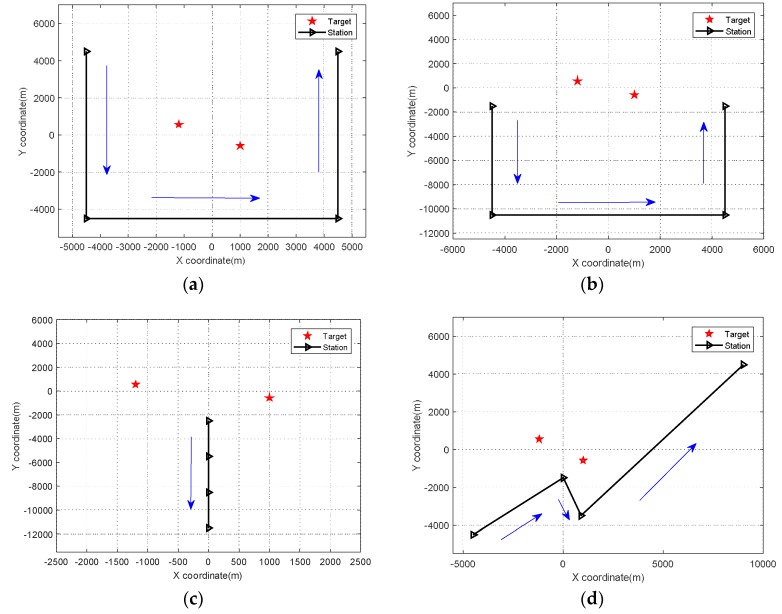
Trajectory of observation movement. (**a**) trajectory around the target sources (**b**) trajectory on one side of the target sources. (**c**) trajectory along the axis of the target sources (**d**) one of the arbitrary movement trajectories.

**Figure 12:**
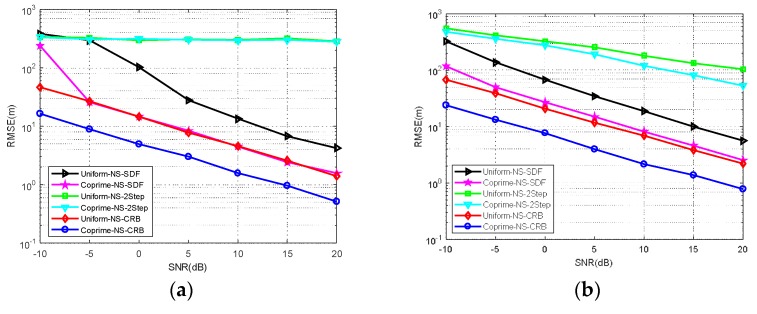

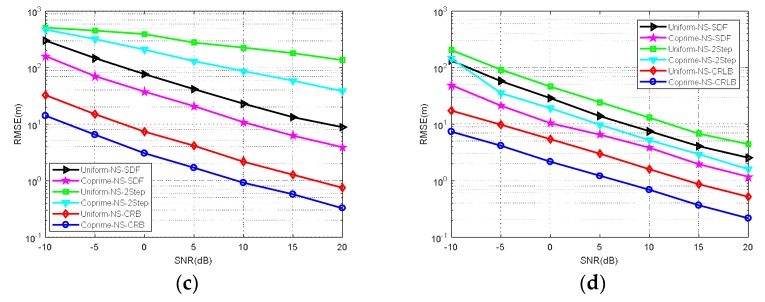
RMSE comparison under the four trajectories shown in Figure 11. (**a**) under the trajectory around the target sources (**b**) under the trajectory on one side of the target sources. (**c**) under the trajectory along the axis of the target sources (**d**) under one of the arbitrary movement trajectories.

The observation position is another important factor affecting the RMSE of position determination. In this simulation, the RMSE performance was examined under four typical trajectories (see Figure 11). The results are shown in Figure 12. From the simulation results, we can see that two-step localization is more sensitive than DPD to the observation location, and the RMSE performance of MCA-DPD varies according to the trajectory, as does the CRLB. This is because the array manifold in the CRLB expression contains the location information of the observation station. Thus, the RMSE performance is affected by the observation position. The estimation accuracy is also affected by the direction of arrival to the linear array in the two-step localization algorithm. Generally, a direction of arrival closer to the normal results in higher accuracy. The MCA-DPD algorithm is also established on the basis of this angle, as in Equation (4), so the RMSE of the proposed method is affected by the observation position.

**Simulation** **5.** *RMSE comparison of MCA-DPD, SDF, two-step localization, and the CRLB for different modulation signals.*

**Figure 13:**
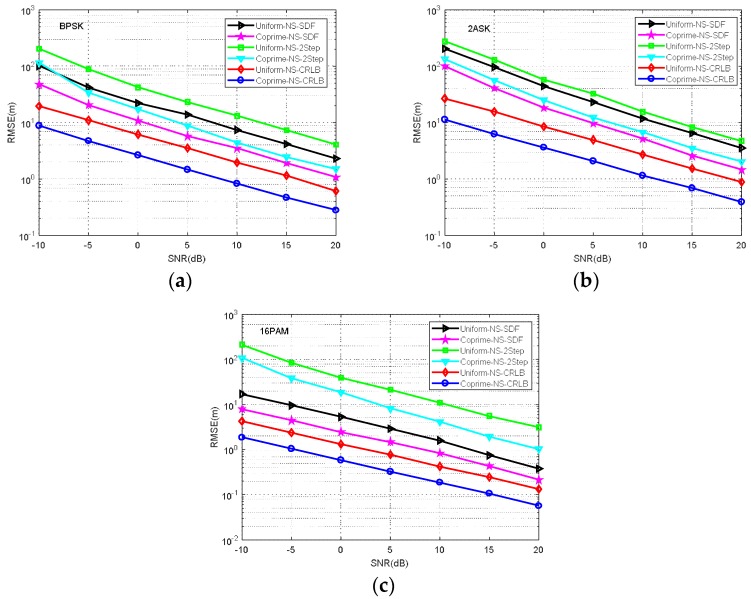
RMSE comparison of different modulation signals (NS). (**a**) BPSK (**b**) 2ASK (**c**) 16PAM.

The signal modulation is known to affect the positioning accuracy. To verify the influence of different modulation signals, we compared the algorithms’ performance under three typical modulation signals (BPSK, ASK, 16PAM) in the NS case. The results in Figure 13 show that the RMSE performance varies according to the modulation signal, as does the CRLB.

## 6. Conclusions

To overcome the problem of insufficient estimation precision for SDF, this study developed an MCA-DPD algorithm that can effectively improve the multi-target positioning precision of DPD. This paper has described a sparse coprime array model for DPD, and demonstrated the improved DPD performance for multiple non-circular sources with a moving array via the high DOF and high-precision characteristics of the coprime array. The complexity of MCA-DPD and SDF, based on a uniform linear array, was compared, and the CRLB of MCA-DPD for non-circular sources was derived. Theoretical analysis and simulation results show that, for both circular and non-circular sources, the proposed algorithm achieves higher position estimation accuracy, with a slight increase in complexity.

## Figures and Tables

**Table 1:** Simulation conditions for the experiments.

Simulation Parameters	Value
sensor spacing	M=4, N=5
antenna number	M+N−1=8
source number	D=2
source position	(−1200, 560), (1000, −574)
snapshot number	K=200
observation location number	L=4
carrier frequency	f=2.1 GHz
speed of light	$c=3\times 10^{8}\;m/s$
Monte Carlo times	Q=200
signal-to-noise ratio (*SNR*)	*SNR* = 10~20 dB
